# Effectiveness of rehabilitation intervention in persons with Friedreich ataxia

**DOI:** 10.3389/fneur.2023.1270296

**Published:** 2023-11-02

**Authors:** Gabriella Paparella, Cristina Stragà, Marinela Vavla, Nicola Pesenti, Vasco Merotto, Gian A. Martorel, Sara Zalunardo, Maria Armellin, Jimmy Comiotto, Andrea Martinuzzi

**Affiliations:** ^1^Department of Conegliano, Scientific Institute, IRCCS E. Medea, Treviso, Italy; ^2^Paediatric Neurology and Neurophysiology Unit, Department of Women's and Children's Health, University Hospital of Padova, Padova, Italy; ^3^Revelo Datalabs Srl, Milan, Italy; ^4^Associazione Brain odv – Altavilla Vicentina, Vicenza, Italy

**Keywords:** Friedreich ataxia, inpatients, rehabilitation, treatment outcome, children, adults

## Abstract

**Introduction:**

The relevance of rehabilitation in progressive neurological disorders, such as Friedreich’s Ataxia (FRDA), has yet to be convincingly proven. FRDA is characterized by ataxia, loss of gait, scoliosis, cardiomyopathy, dysarthria and dysphagia, with reduced life expectancy. The disease onset is usually in adolescence, leading to progressive disability. Omaveloxolone has been recently approved as the first pharmacological treatment for FRDA in adults and adolescents aged 16 years and older. Regarding non-pharmacological therapies, neurorehabilitation is a valuable aid in addressing the symptoms and in maintaining the residual functioning. We performed a prospective observational cohort study to evaluate the efficacy of inpatient rehabilitation (IR) for people with FRDA.

**Methods:**

A total of 42 individuals (29 adults and 13 children) with FRDA were recruited. There were 27 ambulant and 15 non-ambulant participants. The patients underwent IR of 3 and 4 weeks in children and adults, respectively. The IR treatment was designed to be applied within a multidisciplinary setting, so FRDA patients underwent, in addition to physiotherapy, also occupational therapy, practical manual activities and psychological support aiming to enhance transferable skills useful in the activities of daily living. The primary outcome was the Scale for the Assessment and Rating of Ataxia (SARA). Other measures were: Friedreich Ataxia Rating Scale (FARS) and Nine Hole Peg Test (NHPT). Furthermore, we used the 6 Minute Walk Test (6MWT), the Timed Up and Go (TUG) and the Berg Balance Scale (BBS) only on ambulant subjects. Outcomes were evaluated at baseline and at the end of the treatment.

**Results:**

We report that the IR significantly improves motor performance and ataxia symptoms in patients with FRDA. Our study shows significant functional improvement in all the outcome measures used, except for NHPT bilaterally. FARS and SARA scores post-IR are significatively reduced when compared (*p* < 0.001).

**Discussion:**

We demonstrate that IR programs in FRDA can provide a meaningful clinical improvement in terms of outcome measures. These findings could be useful when approaching progressive neurological disorders.

## 1. Introduction

Friedreich’s ataxia (FRDA) is an autosomal recessive neurodegenerative disorder. It is caused by an intronic GAA triplet repeat expansion in the *FXN* gene on chromosome 9 (1). Frataxin is a mitochondrial protein involved in iron homeostasis. The decreased frataxin production determines increased mitochondrial iron and free radicals leading to cellular damage and death, mostly in cardiac muscle, pancreatic islet cells and the nervous system (2). The age of onset is typically within the first two decades of life and individuals are wheelchair-bound 10–15 years after disease onset (3). FRDA is characterized by a progressive gait and limb ataxia, dysarthria, upper (UL) and lower limb (LL) areflexia, decreased vibration sense and LL muscular weakness. There are also non neurological signs such as scoliosis, pes cavus, hypertrophic cardiomyopathy and diabetes mellitus. Often, scoliosis and pes cavus can anticipate the neurological signs (3, 4). Age of onset, progression and severity are not uniformly distributed across patients, but variably correlate with the short allele expansion size (3, 5, 6). Treatment options are currently very limited for FRDA patients. In early 2023 Omaveloxolone has been approved as the first and only pharmacological treatment for FRDA in adults and adolescents aged 16 years and older. It is capable of improving modified Friedreich Ataxia Rating Scale (mFARS) score after chronic treatment (7–9).

Movement in humans is based on the continuous interaction between the individual, the task and the environment (10). The motor skills learning is more concentrated on achieving goals rather than acquiring specific movements. Therefore, the acquisition and adaptation of motor skills involves processes associated with practice and experience. The cerebellum, involved in the interface between the sensorimotor and cognitive control domains, has a direct role in the coordination, shape and fine control of movement and cognitive functions (11). It integrates sensory inputs, mainly visual, proprioceptive and vestibular, with voluntary motor action in order to ensure timing, duration and amplitude of the muscle activity (12). The cerebellum also plays a fundamental role in internal models for the adaptation of the motor gesture during a task (feed-forward control) and motor-learning (11–13). Accordingly, individuals with cerebellar ataxia present impairments in motor learning regarding both simple and complex motor skills, which can limit functional recovery. For this reason, physiotherapy treatment in patients with degenerative ataxia has long been the subject of discussion (12, 13).

Neurorehabilitation is recognized as one of the most useful non-pharmacological therapies to clinically improve cerebellar disorders involving balance, gait ataxia, UL and LL incoordination and postural sway (14–20). The majority of studies have been carried out with patients with spinocerebellar ataxia, and very few have been performed in the FRDA population. These studies present a significant heterogeneity with respect to characteristics of the participants, outcome measures, the training type, intensity and duration of rehabilitation and the settings of interventions.

Milne et al. (21) performed an interesting systematic review of rehabilitation interventions for subjects with degenerative genetic ataxia. Out of a total of 292 subjects involved, only 67 were affected by FRDA. Seven types of intervention were proposed in the examined studies: coordination and balance training, multifaceted inpatient rehabilitation, a cycling regime, balance training, treadmill training, occupational therapy and inspiratory muscle training. Several settings were contemplated: inpatient, outpatient and home-based.

Only few studies have considered a multidisciplinary inpatient approach (16, 20, 22). Among them, the work of Miyay et al. (22) that described in detail the combined treatment of occupational therapy and physiotherapy, even if on patients not FRDA, is very interesting.

Milne et al. (21) state that “coordination and balance training and multifaceted inpatient rehabilitation demonstrated the greatest percentage change in the SARA, while multifaceted inpatient rehabilitation appeared to have the greatest impact on function.” Additionally, “preliminary findings suggest that multifaceted programs incorporating more than one focus, such as coordination and balance training or multidisciplinary inpatient programs, may have greater effect than singularly focused therapies such as balance training or occupational therapy alone.”

Considering intensity, some authors have pointed out that the effectiveness of interventions is also correlated with the intensity of exercise (15, 17). Regarding the duration, improvements in ataxia apparently require a minimum of 4 weeks (21).

The majority of multidisciplinary work involved only patients aged 15 years or older. A systematic review by Hartley et al. (23) concluded that the studies analyzed show promising results but no definitive conclusions could be drawn on the effectiveness of exercise and physical therapy in children.

The published literature so far provides detailed protocols related to the physiotherapy program only (19, 24). These protocols focus not only on balance and coordination, but also on muscle strengthening and proprioception. In light of the previous studies, we hypothesized that multidisciplinary intensive IR could be beneficial to FRDA, and perhaps modify disease progression. Such setting would allow also for minimizing possible environmental confounders and thus it was deemed as the most appropriate for a prospective study.

According to the International Classification of Functioning, Disability and Health (ICF) (25), health is the product of the interaction of the contextual factors with the individual functioning and disability is defined as a negative interaction among these factors. Thus, following the bio-psychosocial model of the ICF it is possible to obtain the information on health starting from the evaluation of body functions and structures, activities and participation, but necessarily considering how the contextual factors modulate the individual functioning in the various situations of life. The activities are the execution of a task or set of actions by an individual and participation is the involvement in a life situation. The importance of the role of occupations and the close relationship between the person, activity and the environment emerge (26). Our goal is not only to work on function, but also on activity and participation optimizing in the same time the contextual factors.

Therefore, we designed an experiment aiming to assess, by means of standardized ataxia outcome measures, the efficacy of neurorehabilitation as IR in FRDA, in patients with different age range and disease severity. The second aim was to create and test a specific IR protocol tailored to the specific needs and functional disabilities in FRDA.

## 2. Materials and methods

### 2.1. Design

**Figure 1:**
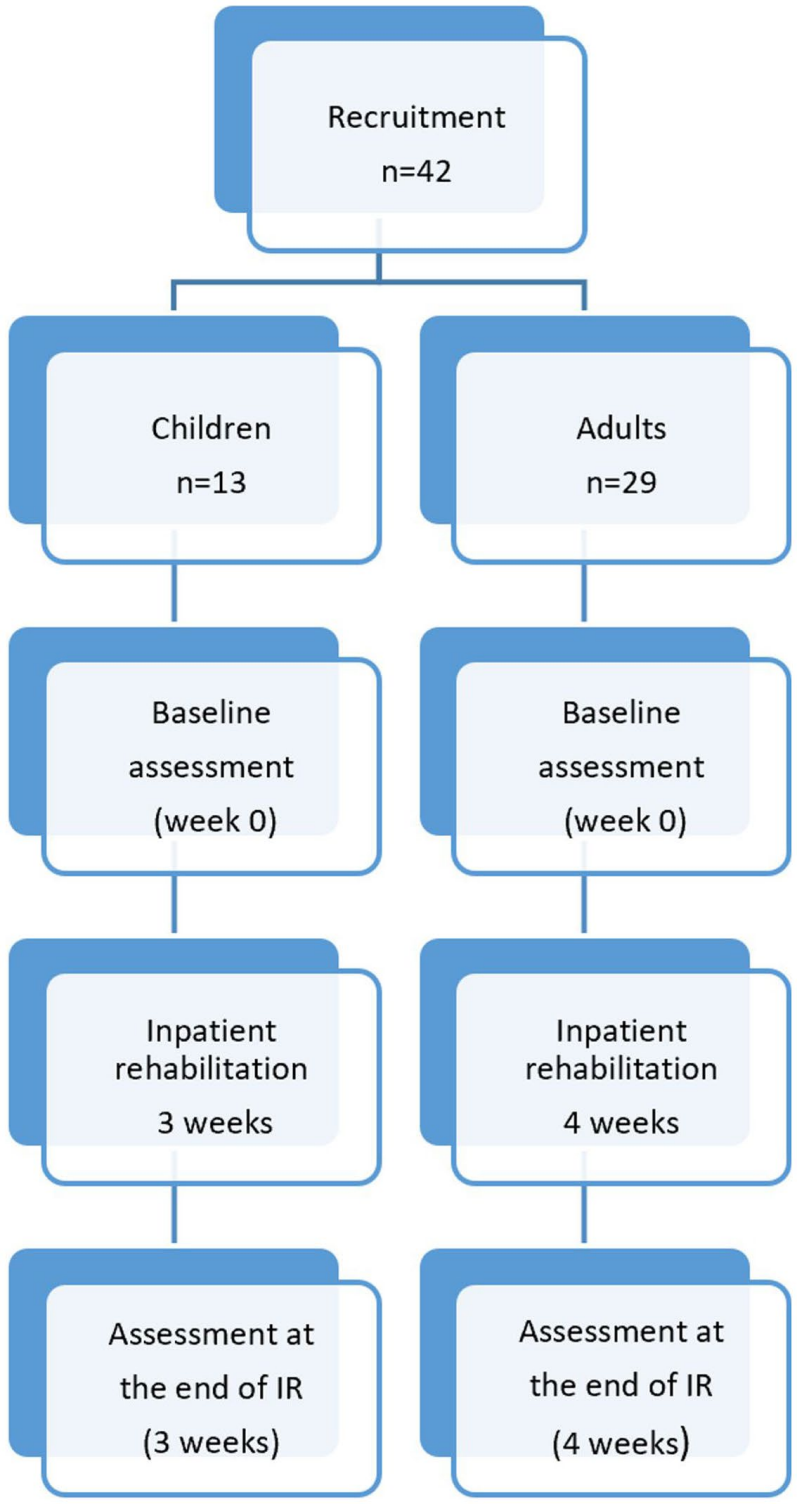
Study scheme. IR, inpatient rehabilitation.

This was a prospective observational cohort study, as classified by the Institutional Review Board (IRB). The study scheme is presented in Figure 1.

### 2.2. Participants

A total of 42 individuals with FRDA were recruited at the “Eugenio Medea” Scientific Institute in Pieve di Soligo and Conegliano (Treviso, Italy) to take part in this study. All participants and their parents/legal tutors were informed regarding the experimental nature of the study and signed the informed consent in accordance with the Declaration of Helsinki (World Medical Association, 1964). The study was reviewed and approved by the competent Ethics Committee (Prot. No 051/11-CE).

The inclusion criteria were: genetic diagnosis of FRDA with GAA trinucleotide expansion or gene mutation, willingness to participate in the study. Exclusion criteria included contemporary participation in other clinical trials or introduction of new disease modifying treatments; any other form of physiotherapy external to the study protocol, any acute condition or orthopedic injury that could eventually limit the ability to fully participate in the intensive therapy schedule.

### 2.3. Intervention

The patients underwent an IR protocol created at the study center *ad hoc* for FRDA patients, considering their main needs and disability impairments as identified by the appropriate ICF categories and qualifiers (Table 1).

**Table 1:** Summary of the IR study protocol.

**IR**	**ICF**	**Goal treatment**	**Physiotherapy program**
First phase	Function	Improve balance and postural reaction.	Improve sensory-motor afferents. Stimulation of the foot-core. Dynamic stability. Timing of activation. Static and dynamic balance.
Second phase	Activity	Increase performance and safety in ADL (locomotion and/or postural passages).	Stand up/sit down training. Go up/down the stairs. Gait training. Transfer practice.
Third phase	Environmental Factors	Mobility aid and orthosis identification.	Training in the use of aids. Consultation with a specialized orthopedic technician.

IR, inpatient rehabilitation; ICF, international classification of functioning, disability and healthy; ADL, activities of daily living.

The FRDA program, based in an individualized IR ward, consisted of a multidisciplinary management (physiotherapy, occupational therapy, practical manual activities, psychological support, speech therapy, clinical psychology). Our goal was to focus not only on function but also on activity and participation by modulating environmental factors.

The IR duration was set to be of 3 and 4 weeks in children and adults, respectively. Literature shows that a period of intensive rehabilitation of at least 4 weeks can improve the function in FRDA (21). Therefore, we chose a 4 weeks duration IR for the adults and 3 weeks duration IR for the children cohort. The latter group duration was tailored in relation to their maximal compliance abilities. All patients received 11 weekly physiotherapy sessions of 45 min each (2 daily sessions from Monday to Friday and 1 session on Saturday morning).

The focus of the physiotherapy program was to improve physical abilities, in particular trunk stability and postural control with activation of deep trunk musculature. This was performed conveying the correct sensitive information, performing the accurate stimulation of the anticipatory postural adjustments (APAs) that would eventually lead to a reduction of the synergism and compensations of postural muscles, and furthermore an adequate orientation of proximal segments and finally distal coordination.

The physiotherapy program was goal-related and specifically designed in accordance with the level of clinical severity. The cohort was divided into two groups: ambulant and non-ambulant. Based on the staging of the Friedreich’s Ataxia Rating Scale (FARS), ambulant patients were considered to be those able to walk, even with any type of aid (FARS stage ≤4.0). Non-ambulant patients were the ones confined to wheelchairs (27). The former underwent program oriented to improve balance and gait pattern, subsequently preventing falls and inactivity. The latter underwent program oriented to trunk control, ULs motility and training of safer and more functional transfer techniques.

Physiotherapy was designed to be applied within a multidisciplinary setting. Therefore, FRDA patients also underwent occupational therapy, practical manual activities and psychological support aiming to enhance transferable skills useful in the activities of daily living (ADLs). In particular, occupational therapy and practical manual activities were useful to strengthen and maintain the skills acquired in the physiotherapy setting.

Patients performed 4 occupational therapy sessions of 45 min each per week. Occupational therapy goals are highly individualized and are set to help individuals improve their ability to perform daily activities and enhance their quality of life. The occupational therapy program empathized coordinative task of the upper limbs and trunk and dual-motor task such as handling objects while standing and walking.

Adult subjects also performed practical manual activities (6/week of 45 min each). The activity consists in carrying out laboratory activities (e.g., construction of wooden products, drawings, mosaic compositions, weaving of wicker baskets). Such activities can be carried out sitting or standing, depending on the severity of the patient. For the children, these activities were integrated into occupational therapy.

All patients received psychological support (3/week of 45 min each) for disease acceptance and elaboration of their experience, for coping strategy and acceptance of adaptations and aids identified by the physiotherapist and occupational therapist.

Moreover, in the first week of hospitalization, all patients underwent neuropsychological assessment and speech therapy in 5 and 3 sessions (45 min each) respectively. The speech therapist evaluated swallowing and language.

The detailed protocol of interventions is illustrated in Table 2.

**Table 2:** Detailed protocol of interventions.

Intervention	Frequency (/Week)	Time/Session	Hours/Week	Type	Modality	Goals/Targets
Physiotherapy	11	45′	8.15	Individual	Foot stimulation Manual facilitation Verbal facilitation Controlled work posture (kneeling position, kneeling knight position, on all fours, normal stance and one-leg stance) Controlled work setting using stable point (tables, wall, beds) Dual task exercises	Improved correct sensory-motor afferences Improved trunk stability and postural control Improved balance Improved upper limb motility Training of safer and more functional transfer techniques Improved gait pattern Prevention of falls and inactivity Identification of mobility aid and orthoses
Occupational therapy	4	45’	3	Individual	Carrying out daily life activities in an ecological environment to encourage the consolidation and generalization of the motor skills treated by the physiotherapist. Simulation of domestic activities in ecological context (kitchen, bedroom, bathroom)	Improved fine motor skills Increased mobility and balance Pain management Energy conservation Enhanced independence Adaptive equipment training Work and school support Enhanced quality of life
Practical manual activities	6	45’	4.30	Individual	Carrying out bi-manual activities (e.g., construction of wooden artefacts, drawings, mosaic compositions, weaving of wicker baskets) both in a standing and sitting position (depending on the severity of the illness). Transport of instruments within the laboratory Controlled work setting (proper work posture, adequate high table, appropriate seating)	Improved UL coordination Improved fine motor skills Improved trunk stability and postural control Increased mobility and balance Improved organization of work setting
Speech therapy	3 (1st week)	45’	2.15	Individual	Speech therapy in the study and possibly during lunch	Evaluation of swallowing and language
Psychological support	3	45’	2.15	Individual	Meetings with the patient Meeting with family members	Emotional well-being Behavioral change Enhanced coping skills Acceptance of adaptations and aids Improved relationships Enhanced quality of life
Neuropsychology	5 (1st week)	45’	3.45	Individual	Administration of the battery of neurocognitive tests	Neuropsychological assessment

### 2.4. Outcome measures

Patients were clinically assessed according to a specific clinical protocol administered by trained and expert physiotherapists. The assessing physiotherapists were not directly involved in the treatment sessions. The assessments were taken at the same time of the day for both pre-IR and post-IR assessment.

The primary outcome was the Scale for the Assessment and Rating of Ataxia (SARA) (28) that is based on a semi-quantitative clinical assessment of cerebellar ataxia (spinocerebellar, Friedreich’s and sporadic ataxia) on an impairment level. SARA has 8 items (gait, stance, sitting, speech, finger-chase, nose-finger, fast alternating movements, heel-shin). It has a maximum score of 40 (severe ataxia) and a minimum of 0 (no ataxia).

Other measures included were the Friedreich Ataxia Rating Scale (FARS) (27) that is a disease-specific outcome measures comprising a functional ataxia staging score, activities of daily living (ADL) subscale, a neurological exam (bulbar, upper limb coordination, lower limb coordination, peripheral nervous system and upright stability) and instrumental tests such as Nine Hole Peg Test (NHPT) (29) to assess hand dexterity bilaterally. NHPT is the most commonly used tool for assessing manual dexterity. It consists of a plastic console with a round dish for the pegs at one end and a nine-hole peg-board at the opposite end. Subject must take pegs from a container, one by one, and insert them into the holes in the board, as quickly as possible. Then the patient has to remove the pegs from the holes, one by one, and put them back in the container. The total time to complete the activity is recorded. Both the dominant and non-dominant hand are tested twice. The Friedreich Ataxia Rating Scale neurologic examination (nFARS) consists of 5 subscales directed to bulbar function (maximum of 11 points), upper limb coordination (maximum of 36 points), lower limb coordination (maximum of 16 points), peripheral nervous system (maximum of 26 points), and upright stability (US; maximum of 28 points). The neurological exam has a maximum score of 117 (severe ataxia). The staging systems used in ataxia are mainly based on walking disability. The score ranges between 0 (normal) to 6 (confined to wheelchair or bed with total dependency for all activities of daily living. Total disability).

Furthermore, only on ambulant subjects, we used the 6 Minute Walk Test (6MWT) (30, 31) to measure the ambulation skills and the Timed Up and Go test (TUG) (32) that assesses mobility, balance, walking ability, and fall risk. The equilibrium was assessed by the Berg Balance Scale (BBS) (33) (range 0–56), a 14-item validated scale that assesses balance abilities during sitting, standing and positional changes (scores of ≤43.5 suggest risk of falls).

### 2.5. Statistical analysis

Patient characteristics were summarized using descriptive statistics. Mean (SD), median (IQr) were used for continuous variables with normal and non-normal distribution, respectively. Absolute frequency (Percentage) for categorical variables.

To evaluate the intervention effect pre-post we used paired *T*-test or Wilcoxon signed rank test based on the normality of the distribution. Comparison between the extent of improvement for the outcome measures between different groups (ambulant vs. non-ambulant subjects and adults vs. children), independent *t*-test or Mann–Whitney *U* test were used.

Spearman’s correlation coefficient was used to study the correlation between disease duration or the number of triplets and the extent of improvement.

All tests were two-tailed and *p* < 0.05 was considered significant. All statistical analyses were performed using R version 4.0.1 (R Foundation for Statistical Computing, Austria) (34).

## 3. Results

### 3.1. Patients

According to defined inclusion criteria we recruited 42 patients, 29 adults and 13 children (age range 8–11 years *N* = 2; age range 12–17 years *N* = 11), with an age of 20.7 ± 8.6 (mean ± SD) years and disease duration of 8.9 ± 4.7 years. Patients declared an age at onset of about 11.7 ± 7.7 years. There were 29 females (69%). Thirty-nine of the patients’ cohort were homozygous for the GAA repeat expansion (93%). Three participants had a heterozygous GAA expansion (two presented a 460 GAA repeat expansion on one allele and a nonsense point mutation on the other; one carried a 666 GAA repeat expansion on one allele and a nonsense point mutation on the other). The mean GAA repeat expansion in the short-allele (GAA1) was 701.1 ± 136.8 (range 383–956), while the long allele (GAA2) counted for 911.8 ± 197.5 (range 460–1,436). The mean SARA score was 17.4 ± 7.8 (range 6.0–32.5), therefore patients of variable severity were included. Twenty-seven patients (64%) were able to walk with or without walking aids on a level surface. In particular: 18 of them walk independently (8 adults and 10 children), 5 with a walker, 2 with support (wall or person), 1 with a cane and 1 with crutches.

The demographic and clinical data of patients are presented in Table 3.

**Table 3:** Demographic and clinical data of patients included in this study.

**Variable**	**Patients (*N* = 42)**
**Adults**	29 (69)
Male gender	7 (24)
Age at onset	
*Early*	4 (14)
*Typical*	18 (62)
*Intermediate*	5 (17)
*Late*	2 (7)
**Children**	13(31)
Male gender	6 (46)
Age, range	
*8–11 years*	2 (15)
*12–17 years*	11 (85)
Age at onset	
*Early*	8 (62)
*Typical*	5 (38)
Age, years	20.7 ± 8.6 (8–49)
Age at onset, years	11.7 ± 7.7 (4–44)
Disease duration, years	8.9 ± 4.7 (1–23)
GAA1	701.1 ± 136.8 (383–956)
GAA2	911.8 ± 197.5 (460–1,436)
SARA score	17.4 ± 7.8 (6.0–32.5)
**Hand dominance**	
R	38 (90.5)
L	4 (9.5)
**Ambulant subjects**	27 (64.3)
Adults	16 (59.3)
Children	11 (40.7)
**Ambulation**	
Independent	18 (66.7)
*Adults/Children*	8/10
With a walker	5 (18.5)
With support (wall/person)	2 (7.4)
With a cane	1 (3.7)
With crutches	1 (3.7)
**Non-ambulant subjects**	15 (35.7)
Adults	13 (86.7)
Children	2 (13.3)

Data are presented as Number (percentage) or Mean ± SD (Range). Based on the work of Rummey et al. (35), we have divided the age at onset in early (0–7 years), typical (8–14 years), intermediate (15–24 years), and late (>24 years); GAA1, GAA repeat expansion in the short-allele; GAA2, GAA repeat expansion in the long-allele; R, right; L, left.

### 3.2. Intervention and evaluations

All patients underwent IR, completing the proposed treatment and no adverse event was reported by any of the participants. All patients were evaluated according to the clinical protocol. Non-ambulant patients were not subjected to the following tests: 6MWT, BBS, TUG. Only three patients did not perform the BBS and one the TUG scale as these scales were initially optional.

### 3.3. Variations of scales in time

**Figure 2:**
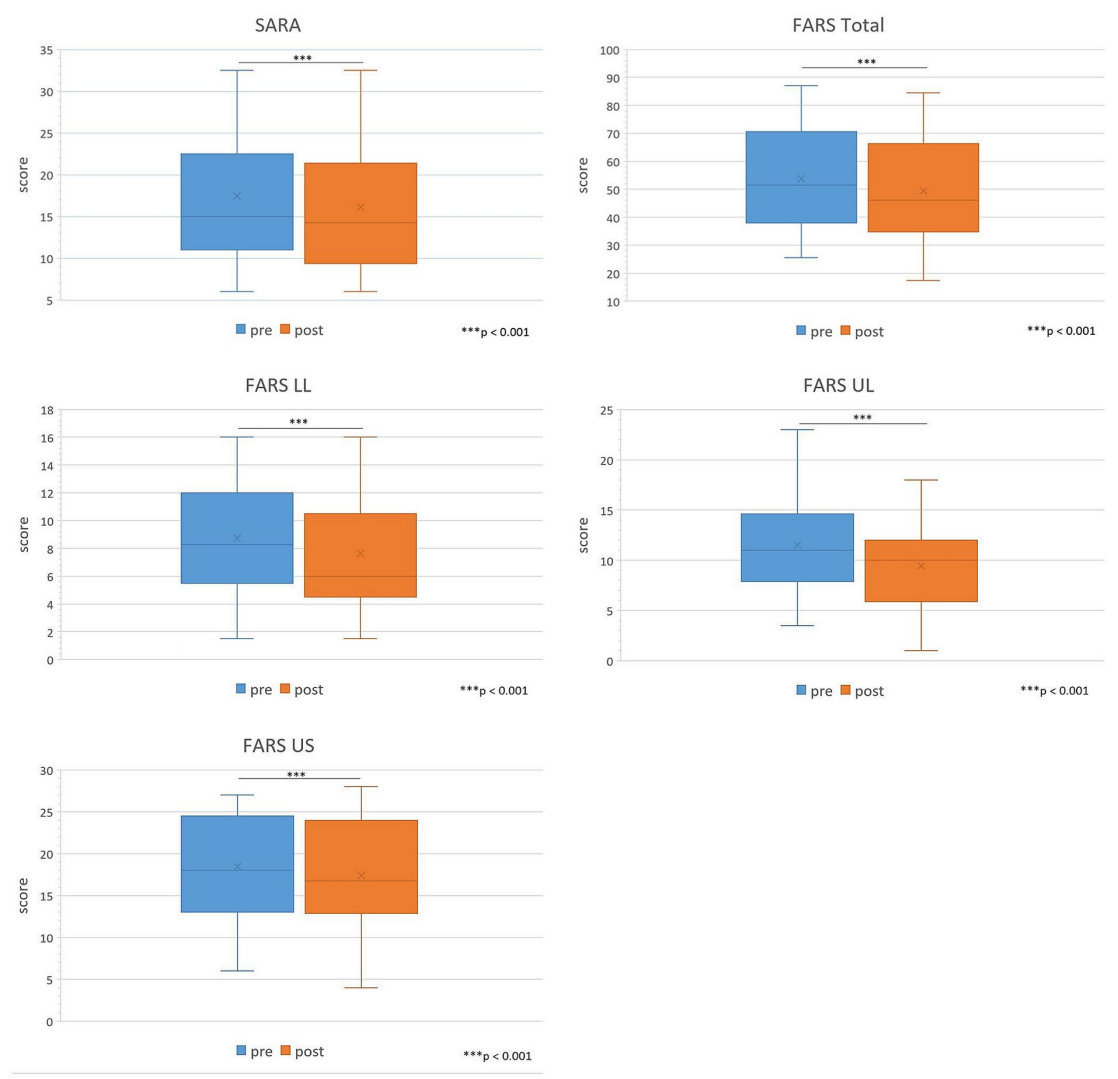
Comparison of SARA, FARS Total, FARS LL, FARS UL and FARS US administered to the entire cohort of patients pre vs. post IR. LL, lower limb; UL, upper limb; US, upright stability. Sig: *** <0.001.

**Figure 3:**
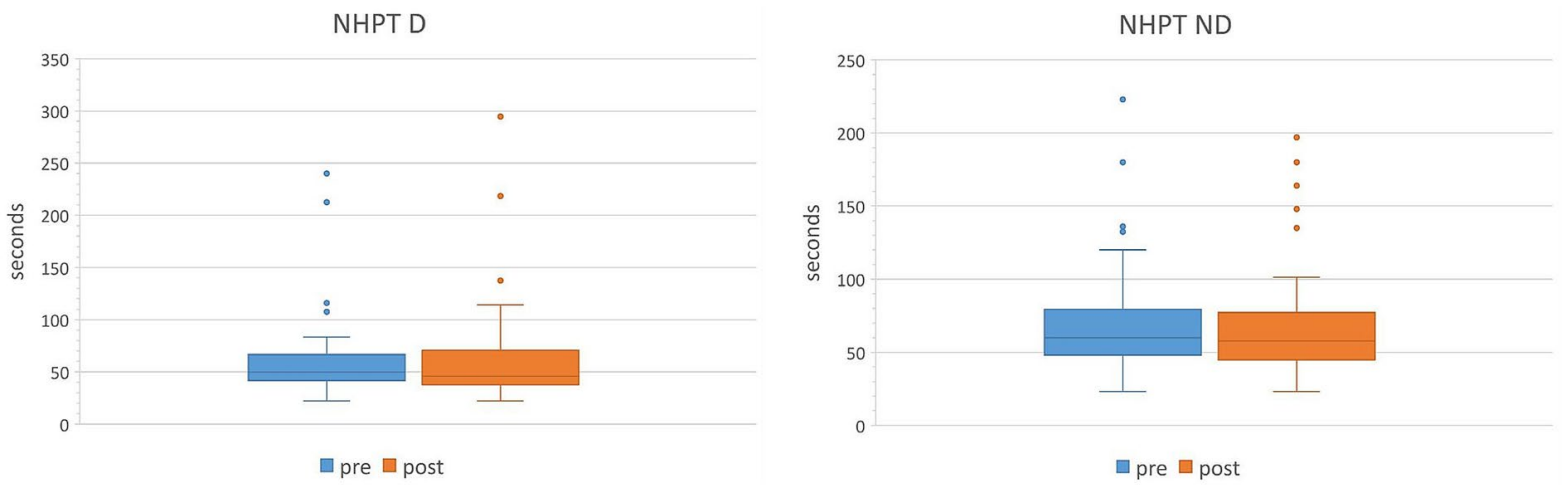
Comparison of NHPT in dominant and non-dominant hand administered to the entire cohort of patients pre vs. post IR. D, dominant hand; ND, non-dominant hand.

Assessments were carried out at the beginning of the IR (pre) and at the end (post). Table 4 presents evolutions of the scales administered to the entire cohort of patients. All patients benefited from rehabilitation treatment in all measured scales. We observed a significant reduction in SARA scale (from 17.4 to 16.1, average reduction of 1.3, *p* < 0.001). Similar significant variations were found also in total FARS scale (average reduction of 4.3, *p* < 0.001) and in all its subscales (average reduction FARS LL of 1.1, *p* < 0.001; FARS UL: 2.1, *p* < 0.001; FARS US: 1.1, *p* < 0.001), as shown in Figure 2. In NHPT there was a reduction in the task execution time but the change was not statistically significant in either the dominant hand or the non-dominant one (see Figure 3).

**Table 4:** Comparison of the scales administered to the entire cohort of patients pre vs. post IR.

	**Pre (*N* = 42)**	**Post (*N* = 42)**	Value of p*
SARA	17.4 (7.8)	16.1 (7.9)	<0.001
FARS total	53.7 (17.2)	49.4 (17.7)	<0.001
*FARS LL*	*8.7 (4.4)*	*7.6 (4.5)*	*<0.001*
*FARS UL*	*11.5 (4.6)*	*9.4 (4.2)*	*<0.001*
*FARS US*	*18.5 (6.0)*	*17.4 (6.7)*	*<0.001*
NHPT (D)	49.7 [42.3, 63.7]	45.7 [38.2, 69.5]	0.121
NHPT (ND)	59.9 [48.4, 78.7]	57.8 [45.3, 75.9]	0.168

Mean (SD) or Median [IQr] are shown. * Value of *p*s from: paired *t*-test [mean (SD)] or Wilcoxon signed-rank test (median [IQR]). LL, lower limb; UL, upper limb; US, upright stability; D, dominant hand; ND, non-dominant hand. The characters in italics, both in the acronyms and in the numbers, have been used to distinguish the 3 subscales of the total FARS scale.

We then divided the cohort into ambulant (*n* = 27) and non-ambulant (*n* = 15) patients (Table 5). In both groups there was a significant improvement in the SARA, FARS Total and its subscales.

**Table 5:** Comparison of the scales pre vs. post IR in ambulant and non-ambulant subjects.

**Ambulant subjects (*N* = 27)**			
	**Pre**	**Post**	Value of p*
SARA	12.4 (3.8)	11.1 (3.7)	<0.001
FARS Total	44.7 (13.1)	39.8 (12.4)	<0.001
*FARS LL*	*6.3 (3.1)*	*5.1 (2.4)*	*<0.001*
*FARS UL*	*10.2 (4.5)*	*8.1 (3.9)*	*<0.001*
*FARS US*	*14.7 (4.0)*	*13.3 (4.4)*	*<0.001*
6MWT	294 [198.9, 353.3]	340 [238.5, 388.5]	<0.001
BBS	38.2 (11.8)	41.7 (11.5)	<0.001
TUG	16.5 (11.1)	14.5 (9.5)	0.007

**Non-ambulant subjects (*N* = 15)**			
	**Pre**	**Post**	Value of p*
SARA	26.4 (4.3)	25.0 (5.2)	0.002
FARS total	69.8 (10.5)	66.6 (11.7)	0.002
*FARS LL*	*13.0 (2.9)*	*12.2 (3.7)*	*0.015*
*FARS UL*	*13.9 (4.0)*	*11.9 (3.6)*	*0.006*
*FARS US*	*25.2 (1.1)*	*24.8 (1.4)*	*0.055*

Mean (SD) or Median [IQr] are shown. * Value of *p*s from: paired *t*-test [mean (SD)]; Wilcoxon signed-rank test (median [IQR]). *N* = 24 in BBS and *N* = 26 in TUG. LL, lower limb; UL, upper limb; US, upright stability. The characters in italics, both in the acronyms and in the numbers, have been used to distinguish the 3 subscales of the total FARS scale.

We then compared the extent of improvement between the two groups: ambulant vs. non-ambulant (Supplementary Table S1). No significant differences emerged, except for FARS US (average reduction of 1.4 in ambulant vs. 0.3 in non-ambulant subjects, *p* = 0.008, independent *t*-test).

**Figure 4:**
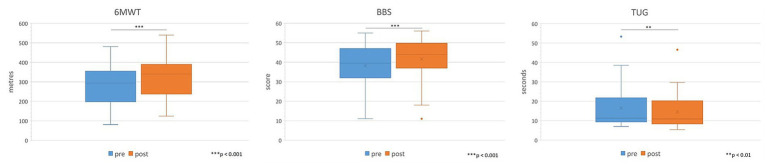
Comparison of 6MWT, BBS, and TUG administered only to ambulant patients pre vs. post IR. Sig: *** <0.001, ** <0.01.

Regarding the assessments performed only on ambulant patients (see Figure 4), in the 6MWT there was a significant variation in terms of increase in meters walked after IR (average increase of 24.5 meters, *p* < 0.001), in the BBS a significant increase in the score (from 38.2 to 41.7, *p* < 0.001) and in the TUG a significant decrease in time spent to perform the task (average reduction of 2 s, *p* = 0.007).

The total cohort was then divided into adults (*n* = 29) and children (*n* = 13). In both groups taken separately the results already seen above were confirmed (Table 6). Regarding NHPT, in both groups there was a reduction in the task execution time but not statistically significant in either the dominant hand or the non-dominant hand.

**Table 6:** Comparison of the scales pre vs. post IR in adults and children.

**Adults (*N* = 29)**			
	**Pre**	**Post**	Value of p*
SARA	19.1 (8.2)	18.0 (8.4)	<0.001
FARS Total	57.1 (18.2)	53.4 (19.0)	<0.001
*FARS LL*	*9.2 (4.9)*	*8.5 (4.9)*	*0.001*
*FARS UL*	*11.3 (4.0)*	*9.4 (4.3)*	*<0.001*
*FARS US*	*19.7 (5.8)*	*18.8 (6.7)*	*0.001*
NHPT (D)	57.2 [46.8, 78.5]	56.5 [41.3, 75.5]	0.883
NHPT (ND)	63.0 [51.3, 90.5]	61.8 [47.3, 91.0]	0.474
6MWT	238 [177.0, 351.2]	284 [185.2, 371.2]	0.002
BBS	36.1 (13.7)	39.6 (13.7)	<0.001
TUG	21.0 (12.7)	18.7 (10.5)	0.061

**Children (*N* = 13)**			
	**Pre**	**Post**	value of p*
SARA	13.7 (5.6)	11.9 (4.8)	<0.001
FARS Total	46.1 (12.1)	40.4 (9.8)	0.002
*FARS LL*	*7.6 (3.0)*	*5.7 (2.4)*	*0.002*
*FARS UL*	*11.9 (6.0)*	*9.5 (4.1)*	*0.007*
*FARS US*	*15.6 (5.7)*	*14.2 (5.6)*	*0.005*
HPT (D)	*43.5 [40.4, 48.9]*	*39.1 [35.8, 45.0]*	*0.087*
NHPT (ND)	48.0 [44.0, 60.0]	46.2 [39.2, 50.4]	0.296
6MWT	319 [290.2, 394.5]	360 [316.5, 402.5]	0.013
BBS	41.7 (7.3)	45.2 (5.9)	0.001
TUG	10.4 (3.7)	8.8 (3.3)	0.004

Mean (SD) or Median [IQr] are shown. * *p*-values from: paired *t*-test [mean (SD)]; Wilcoxon signed-rank test (median [IQR]). Adults: *N* = 16 in 6MWT, *N* = 15 in BBS and TUG; Children: *N* = 11 in 6MWT and TUG, *N* = 9 in BBS. LL, lower limb; UL, upper limb; US, upright stability; D, dominant hand; ND, non-dominant hand. The characters in italics, both in the acronyms and in the numbers, have been used to distinguish the 3 subscales of the total FARS scale.

The comparison between the two groups (adults vs. children) in terms of extent of improvement (Supplementary Table S2) showed no statistically significant difference, except for FARS LL (average reduction of 0.7 in adults vs. 1.9 in children, *p* = 0.012, independent *t*-test).

### 3.4. Correlation

We also assessed the correlation between the extent of improvement and the number of GAA1 triplets repeats and disease duration, respectively. This analysis was performed by considering the whole population and for individual subpopulations of adult/children, ambulant/non-ambulant. No significant results were found.

## 4. Discussion

The aim of this study was to evaluate the efficacy of neurorehabilitation intended as IR in FRDA, in patients with different age range and disease severity, by using standardized ataxia outcome measures. The second aim was to validate an IR protocol tailored to the specific needs and functional disabilities in FRDA, that takes into account not only functions but also the domains of activity and participation as defined by the ICF.

The size of our participants’ sample, albeit not large, is still comparatively one of the most numerous in the literature on FRDA cohorts, since most studies involved small cohorts of non-homogeneous ataxias.

Although there are clinical outcomes validated for FRDA, such as FARS, mFARS and SARA (35), in the literature there is a heterogeneity of indicators, with consequent difficulty in comparing the results of the various studies.

This study is the first to involve a cohort of individuals with FRDA of a wide range of ages and severity, applying a unique assessment protocol. This made it possible to compare the effectiveness of the rehabilitation process between groups of different ages (children and adults) and of different severity (ambulant and non-ambulant subjects).

We show that an inpatient intensive rehabilitation significantly improves motor performance and ataxia symptoms in people with FRDA. Our study shows significant functional improvement in all the outcome measures used, except NHPT bilaterally, even if there was an average reduction of the task execution time. In children, however, the reduction is more marked than in adults, but it must be considered that there is a great variability and a low number of subjects.

The results show a significant reduction in ataxia symptoms measured with the SARA scale. These results are in agreement with several previous studies which have used the same outcome measure to assess efficacy of rehabilitation (14, 15, 17, 18). In our study we had an average improvement of 1.3 points on the SARA scale. This improvement is significant, given that 1 SARA point would be the expected disease progression in 1 year (36). When considered separately, the disease progression is twice as faster in ambulatory FRDA patients compared to non-ambulatory ones (37) and progresses more in early onset patients compared to typical or late onset (35). If these data of natural history are projected on our results that show comparable improvement in all cohorts irrespective of ambulatory status or age of onset, the difference in observed versus expected outcome would highlight a much greater impact of IR on ambulatory and early onset subjects. This observation is not unexpected, since the functioning reserves should be comparably greater in these patients.

In the works of Ilg et al. (14) and Schatton et al. (17) there was reported a variation of the SARA score of 2.5 and 5.2, respectively. However, their cohorts involved patients with different cerebellar ataxia, including FRDA patients. As the same authors underline, patients with afferent ataxia, such as FRDA, show minor improvements compared to subjects with cerebellar ataxia.

In our work we recorded an improvement in UL coordination, with a reduction in the NHPT task execution time but the change was not statistically significant in either the dominant hand or the non-dominant hand. Leonardi et al. (18) has reported a NHPT improvement in the dominant hand in their cohort. Nevertheless, Leonardi’s work has involved a small number of adult ambulant patients with ataxias of various origins and a short disease duration when compared to our cohort.

Disease progression in subjects with FRDA is measures using rating scales, such as SARA and FARS. They represent a valid tool for monitoring the progress of the disease although they depend greatly on axial functions, balance and gait (35% in SARA and 34% mFARS). This varies the sensitivity of the scales to the progression of symptoms in different disease phases. In the ambulatory phase, the worsening is correlated primarily with the mFARS US score and then with the items relating to the LL score (35). The study by Reetz et al. (37) shows that, after the loss of walking, the SARA items relating to the functions of the trunk and lower limbs are affected by the ceiling effect with reduced ability to highlight disease progression. Our study considers ambulatory and non-ambulatory patients. With the same scales used, despite the reduced sensitivity of the SARA and FARS scales in non-ambulatory subjects, we show that subjects with advanced pathology (non-ambulant subjects) also benefit from the rehabilitation program, as already highlighted by Schatton et al. (17) and Milne et al. (19). In the work of Miyai et al. (22), there was greater improvement in mildly affected subjects and this could be due to the fact that they recruited adult ambulatory patients with spinocerebellar ataxia and idiopathic cerebellar ataxia. This result is not confirmed by our study. In the comparison between the two groups (ambulant and non-ambulant), a statistically significant difference was found only in FARS US in the ambulant patients. This datum is an expected result as the items concerning the FARS US reflect the ability to maintain an upright position, balance in tandem and walking. These items are not applicable in the majority of non-ambulant patients.

Although the literature does not confirm the validity of rehabilitation in children (23), our study shows that even in the early stages of the disease we can document benefits. We obtained a significant improvement in LL coordination in children but no changes in walking outcomes of a similar magnitude. Coordination of the LL is a function, while walking represents an activity. Walking is influenced by several factors. In addition to the coordination of the LL, the postural stability of the trunk, weakness and proprioception of the LL are affected. Therefore, improving coordination alone does not automatically change walking.

By comparing adults with children, we demonstrate that the improvement in FARS LL subscale is more significant in children that underwent rehabilitation versus the adults. Observing the entry SARA scores, children involved in the study presented a mild condition and only two patients were non-ambulant. The initial stage of the disease is characterized by a weakness of the muscles for the pelvic stabilization, in particular the gluteus medium and gluteus maximus which, as the disease progresses, involves a greater number of muscles of the lower limbs (38–40). Therefore, in adult patients there is greater impairment of the lower limbs both in terms of weakness, proprioception and coordination. This could explain the difference between the two groups.

In this study we demonstrated the effectiveness of intensive rehabilitation in FRDA individuals with different age range and disease severity. Nevertheless, there are still many questions to address. What is the rationale for an optimal rehabilitation in the context of a progressive neurodegenerative condition? Which is the most efficient frequency and duration of the training sessions? When is the best timing to start with physiotherapy? Which training methods and mechanisms are most useful? How should we adapt the training program to the different stages of the disease and ages at onset?

Our work has confirmed that rehabilitative training leads to improved motor performance. The lack of a control group does not allow us to draw absolute conclusions, both in terms of intensity and in terms of setting.

A multidisciplinary management including occupational therapy (22) is considered appropriate in order to integrate the rehabilitation program into daily practice. Inclusion of occupational therapy in the program was a good motivational support and resulted in improvements in selected areas of activity hardly captured by the reported scales. The experience of the direct transferability of the selected motor improvements in changes in the way tasks of the daily life are carried out results in a strong positive feedback for patients and caregivers.

Our experience shows that also psychological support is a fundamental element in the rehabilitation process. Each patient presents a cognitive-behavioral modality when facing the disease. Each individual has a specific and unique “coping style.” Psychological support represents a moment of listening and elaboration of the experience related to the disease. The psychologist helps to accept the disease and at the same time adhere to the rehabilitation program including the adoption of prescribed aids.

In our study, we expected children to respond and perform better than adults. On the contrary no difference could be demonstrated between the two groups. This could be due to a lack of motivation for intensive and continuous physiotherapy in children. Moreover, by analyzing the age of onset of subjects, most children have an early onset, while in adults the majority have a typical onset, causing a different disease progression. It is also true that the cohort of children is smaller and includes children over 8 years of age, so it does not allow us to generalize the results to the entire pediatric population and does not allow us to draw solid conclusions.

On the assumption that there is little motivation of the children to traditional rehabilitation, it is considered useful to introduce a type of training based on whole body-controlled video games (“exergames”) to maximize engagement in the proposed programs. Even though we have only anecdotical experience of such approach, we believe that this technology supported training may represent a valid alternative to the classic rehabilitation program especially for children to improve, through the playful aspect, the motivation and adherence to the protocol.

Our protocol includes a series of exercises designed to promote muscle strengthening and sensory stimulation. Patients with FRDA already present, in the initial stages, weakness of the muscles of the lower limbs, in particular of the pelvis stabilizers, and weakness of the muscles of the trunk, in particular of the abdominal ones. Proprioceptive sensory loss is an important component of FRDA. Sensory stimulation through active and passive mobilization of the foot/ankle (19) or through electrical stimulation (18) determines changes in the clinical picture.

According to Donchin et al. (41), there are different learning mechanisms based on the stage of the disease. We thus should plan a differentiated rehabilitation path between adult and children and in different disease stages.

## 5. Limitations of the study

A major limitation of this study is the absence of a control group.

Although the number of participants in the study is relevant considering that FRDA is a rare disease, the cohort was too small to be stratified according to expansion size and disease duration. This did not allow to identify biological predictors of the rehabilitation program effectiveness.

Another limitation is the lack of follow up and evaluation of the retention time and long-term effects.

Therefore, it is essential that future studies include larger cohorts of participants and a randomized controlled design. A multi-center design, albeit more difficult to manage and prone to inter-center comparability problems, could provide critical information on protocol transferability and generalizability of the results.

## 6. Conclusion

The findings of this study indicate that inpatient multi-disciplinary rehabilitation determines significant improvements in motor performance and ataxic symptoms in people with FRDA with different age range and disease severity.

The rehabilitation approach must not only focus on a single function but on multiple functions and also on activity and participation optimizing in the same time the contextual factors.

Further studies with more numerous cohorts and randomized controlled design would be appropriate to verify the intensity, type and duration of rehabilitation, taking into account the characteristics of individual patients in terms of disease stage and age at onset.

## Data availability statement

The original contributions presented in the study are included in the article/Supplementary material, further inquiries can be directed to the corresponding author.

## Ethics statement

The studies involving humans were approved by Ethics Committee of IRCCS E.Medea. The studies were conducted in accordance with the local legislation and institutional requirements. Written informed consent for participation in this study was provided by the participants’ legal guardians/next of kin.

## Author contributions

GP: Conceptualization, Data curation, Investigation, Methodology, Writing – original draft, Writing – review & editing. CS: Data curation, Writing – original draft, Writing – review & editing. MV: Conceptualization, Writing – review & editing. NP: Formal analysis, Writing – review & editing. VM: Investigation, Writing – review & editing. GM: Investigation, Writing – review & editing. SZ: Investigation, Writing – review & editing. MA: Investigation, Writing – review & editing. JC: Investigation, Writing – review & editing. AM: Conceptualization, Funding acquisition, Project administration, Supervision, Writing – review & editing.

## References

[1] Campuzano V Montermini L Moltò MD Pianese L Cossée M Cavalcanti F. Friedreich's ataxia: autosomal recessive disease caused by an intronic GAA triplet repeat expansion. Science. (1996) 271:1423–7. doi: 10.1126/science.271.5254.1423 8596916

[2] Maring JR Croarkin E. Presentation and progression of Friedreich ataxia and implications for physical therapist examination. Phys Ther. (2007) 87:1687–96. doi: 10.2522/ptj.20060232, PMID: 17911272

[3] Parkinson MH Boesch S Nachbauer W Mariotti C Giunti P. Clinical features of Friedreich's ataxia: classical and atypical phenotypes. J Neurochem. (2013) 126:103–17. doi: 10.1111/jnc.12317, PMID: 23859346

[4] Schulz JB Boesch S Bürk K Dürr A Giunti P Mariotti C. Diagnosis and treatment of Friedreich ataxia: a European perspective. Nat Rev Neurol. (2009) 5:222–34. doi: 10.1038/nrneurol.2009.26, PMID: 19347027

[5] Dürr A Cossee M Agid Y Campuzano V Mignard C Penet C. Clinical and genetic abnormalities in patients with Friedreich's ataxia. N Engl J Med. (1996) 335:1169–75. doi: 10.1056/NEJM199610173351601 8815938

[6] Schöls L Amoiridis G Przuntek H Frank G Epplen JT Epplen C. Revision of the phenotype according to molecular genetics. Brain. (1997) 120:2131–40. doi: 10.1093/brain/120.12.2131, PMID: 9448568

[7] Lynch DR Farmer J Hauser L Blair IA Wang QQ Mesaros C. Safety, pharmacodynamics, and potential benefit of omaveloxolone in Friedreich ataxia. Ann Clin Transl Neurol. (2018) 6:15–26. doi: 10.1002/acn3.660, PMID: 30656180 PMC6331199

[8] Lynch DR Chin MP Delatycki MB Subramony SH Corti M Hoyle JC. Safety and efficacy of Omaveloxolone in Friedreich Ataxia (MOXIe study). Ann Neurol. (2021) 89:212–25. doi: 10.1002/ana.25934, PMID: 33068037 PMC7894504

[9] Subramony SH Lynch DL. A milestone in the treatment of ataxias: approval of Omaveloxolone for Friedreich Ataxia. Cerebellum. (2023). doi: 10.1007/s12311-023-01568-8, PMID: 37219716

[10] Shumway-Cook A Woollacott MH. Motor control: Translating research into clinical practice. 5th ed. Philadelphia: Wolters Kluwer Health/Lippincott Williams & Wilkins (2016).

[11] Koziol LF Budding D Andreasen N D'Arrigo S Bulgheroni S Imamizu H. Consensus paper: the cerebellum's role in movement and cognition. Cerebellum. (2014) 13:151–77. doi: 10.1007/s12311-013-0511-x, PMID: 23996631 PMC4089997

[12] Matilla-Dueñas A Serrano C Ivánovic Y Alvarez R Latorre P Genís D. Novel therapeutic challenges in cerebellar diseases In: Manto M Schmahmann JD Rossi F Gruol DL Koibuchi N, editors. Handbook of the cerebellum and cerebellar disorders. Dordrecht: Springer (2013). 2370–94.

[13] Marsden J Harris C. Cerebellar ataxia: pathophysiology and rehabilitation. Clin Rehabil. (2011) 25:195–216. doi: 10.1177/0269215510382495 21321055

[14] Ilg W Synofzik M Brötz D Burkard S Giese MA Schöls L. Intensive coordinative training improves motor performance in degenerative cerebellar disease. Neurology. (2009) 73:1823–30. doi: 10.1212/WNL.0b013e3181c33adf, PMID: 19864636

[15] Ilg W Schatton C Schicks J Giese MA Schöls L Synofzik M. Video game-based coordinative training improves ataxia in children with degenerative ataxia. Neurology. (2012) 79:2056–60. doi: 10.1212/WNL.0b013e3182749e67, PMID: 23115212

[16] Milne SC Campagna EJ Corben LA Delatycki MB Teo K Churchyard AJ. Retrospective study of the effects of inpatient rehabilitation on improving and maintaining functional independence in people with Friedreich ataxia. Arch Phys Med Rehabil. (2012) 93:1860–3. doi: 10.1016/j.apmr.2012.03.026, PMID: 22484089

[17] Schatton C Synofzik M Fleszar Z Giese MA Schöls L Ilg W. Individualized exergame training improves postural control in advanced degenerative spinocerebellar ataxia: a rater-blinded, intra-individually controlled trial. Parkinsonism Relat Disord. (2017) 39:80–4. doi: 10.1016/j.parkreldis.2017.03.016 28365204

[18] Leonardi L Aceto MG Marcotulli C Arcuria G Serrao M Pierelli F. A wearable proprioceptive stabilizer for rehabilitation of limb and gait ataxia in hereditary cerebellar ataxias: a pilot open-labeled study. Neurol Sci. (2017) 38:459–63. doi: 10.1007/s10072-016-2800-x, PMID: 28039539

[19] Milne SC Corben LA Roberts M Murphy A Tai G Georgiou-Karistianis N. Can rehabilitation improve the health and well-being in Friedreich's ataxia: a randomized controlled trial? Clin Rehabil. (2018) 32:630–43. doi: 10.1177/0269215517736903, PMID: 29072092

[20] Doğan-Aslan M Büyükvural-Şen S Nakipoğlu-Yüzer GF Özgirgin N. Demographic and clinical features and rehabilitation outcomes of patients with Friedreich ataxia: a retrospective study. Turk J Phys Med Rehabil. (2018) 64:230–8. doi: 10.5606/tftrd.2018.2213, PMID: 31453516 PMC6657791

[21] Milne SC Corben LA Georgiou-Karistianis N Delatycki MB Yiu EM. Rehabilitation for individuals with genetic degenerative Ataxia: a systematic review. Neurorehabil Neural Repair. (2017) 31:609–22. doi: 10.1177/1545968317712469, PMID: 28595509

[22] Miyai I Ito M Hattori N Mihara M Hatakenaka M Yagura H. Cerebellar ataxia rehabilitation trial in degenerative cerebellar diseases. Neurorehabil Neural Repair. (2012) 26:515–22. doi: 10.1177/1545968311425918, PMID: 22140200

[23] Hartley H Cassidy E Bunn L Kumar R Pizer B Lane S. Exercise and physical therapy interventions for children with Ataxia: a systematic review. Cerebellum. (2019) 18:951–68. doi: 10.1007/s12311-019-01063-z, PMID: 31392562 PMC6761087

[24] Milne SC Corben LA Roberts M Szmulewicz D Burns J Grobler AC. Rehabilitation for ataxia study: protocol for a randomised controlled trial of an outpatient and supported home-based physiotherapy programme for people with hereditary cerebellar ataxia. BMJ Open. (2020) 10:e040230. doi: 10.1136/bmjopen-2020-040230, PMID: 33334834 PMC7747606

[25] International Classification of Functioning. Disability and health: ICF. Geneva, Switzerland: World Health Organization (2001).

[26] Haglund L Henriksson C. Concepts in occupational therapy in relation to the ICF. Occup Ther Int. (2003) 10:253–68. doi: 10.1002/oti.189, PMID: 14647539

[27] Lynch DR Farmer JM Tsou AY Perlman S Subramony SH Gomez CM. Measuring Friedreich ataxia: complementary features of examination and performance measures. Neurology. (2006) 66:1711–6. doi: 10.1212/01.wnl.0000218155.46739.90, PMID: 16769945

[28] Schmitz-Hübsch T du Montcel ST Baliko L Berciano J Boesch S Depondt C. Scale for the assessment and rating of ataxia: development of a new clinical scale. Neurology. (2006) 66:1717–20. doi: 10.1212/01.wnl.0000219042.60538.92 16769946

[29] Mathiowetz V Weber K Kashman N Volland G. Adult norms for the nine hole peg test of finger dexterity. Occup Therapy J Res. (1985) 5:24–38. doi: 10.1177/153944928500500102 3160243

[30] Guyatt GH Sullivan MJ Thompson PJ Fallen EL Pugsley SO Taylor DW. The 6-minute walk: a new measure of exercise capacity in patients with chronic heart failure. Can Med Assoc J. (1985) 132:919–23. 3978515 PMC1345899

[31] Enright PL. The six-minute walk test. Respir Care. (2003) 48:783–5. PMID: 12890299

[32] Podsiadlo D Richardson S. The timed "up & go": a test of basic functional mobility for frail elderly persons. J Am Geriatr Soc. (1991) 39:142–8. doi: 10.1111/j.1532-5415.1991.tb01616.x 1991946

[33] Berg K Wood-Dauphinee S Williams JI. The balance scale: reliability assessment with elderly residents and patients with an acute stroke. Scand J Rehabil Med. (1995) 27:27–36. PMID: 7792547

[34] R Core Team. R: A language and environment for statistical computing. Vienna, Austria: R Foundation for Statistical Computing (2022).

[35] Rummey C Corben LA Delatycki M Wilmot G Subramony SH Corti M. Natural history of Friedreich's Ataxia: heterogeneity of neurological progression and consequences for clinical trial design. Neurology. (2022) 99:e1499–510. doi: 10.1212/WNL.0000000000200913, PMID: 35817567 PMC9576299

[36] Marelli C Figoni J Charles P Anheim M Tchikviladze M Vincitorio CM. Annual change in Friedreich's ataxia evaluated by the scale for the assessment and rating of Ataxia (SARA) is independent of disease severity. Mov Disord. (2012) 27:135–9. doi: 10.1002/mds.23879, PMID: 22076850

[37] Reetz K Dogan I Hilgers RD Giunti P Parkinson MH Mariotti C. EFACTS study group. Progression characteristics of the European Friedreich's Ataxia consortium for translational studies (EFACTS): a 4-year cohort study. Lancet Neurol. (2021) 20:362–72. doi: 10.1016/S1474-4422(21)00027-2, PMID: 33770527

[38] Beauchamp M Labelle H Duhaime M Joncas J. Natural history of muscle weakness in Friedreich's Ataxia and its relation to loss of ambulation. Clin Orthop Relat Res. (1995) 311:270–5. 7634585

[39] Sival DA Pouwels ME Van Brederode A Maurits NM Verschuuren-Bemelmans CC Brunt ER. In children with Friedreich ataxia, muscle and ataxia parameters are associated. Dev Med Child Neurol. (2011) 53:529–34. doi: 10.1111/j.1469-8749.2011.03931.x 21574990

[40] Corben LA Collins V Milne S Farmer J Musheno A Lynch D. Clinical management guidelines writing group. Clinical management guidelines for Friedreich ataxia: best practice in rare diseases. Orphanet J Rare Dis. (2022) 17:415. doi: 10.1186/s13023-022-02568-3, PMID: 36371255 PMC9652828

[41] Donchin O Timmann D. How to help cerebellar patients make the most of their remaining learning capacities. Brain. (2019) 142:492–5. doi: 10.1093/brain/awz020, PMID: 30810211

