# Supplementary material for: Effectiveness of rehabilitation intervention in persons with Friedreich ataxia
Source: Front Neurol. 2023 Nov 2;14:1270296. doi: 10.3389/fneur.2023.1270296 (PMC10653317; doi:10.3389/fneur.2023.1270296)
Supplement: Supplementary file 1 [file Table_1.pdf]

Supplementary Material

“Effectiveness of rehabilitation intervention in persons with Friedreich ataxia"

Supplementary Table 1. Comparison of the extent of improvement in ambulant and non-ambulant subjects.

	Non-Ambulant subjects (N=15)	Ambulant subjects (N=27)	*p*-value*
**SARA**	-1.40 (1.40)	-1.30 (1.27)	0.809
**FARS Total**	-3.23 (3.32)	-4.93 (3.98)	0.169
FARS LL	-0.80 (1.11)	-1.26 (1.52)	0.310
FARS UL	-1.97 (2.33)	-2.15 (2.07)	0.796
FARS US	-0.33 (0.62)	-1.44 (1.48)	0.008

Mean (SD) or Median [IQr] are shown. * P-values from: paired t-test (mean (SD) or Wilcoxon signed-rank test (median [IQR]). LL, Lower Limb; UL, Upper Limb; US, Upright Stability
