# Supplementary material for: Effectiveness of rehabilitation intervention in persons with Friedreich ataxia
Source: Front Neurol. 2023 Nov 2;14:1270296. doi: 10.3389/fneur.2023.1270296 (PMC10653317; doi:10.3389/fneur.2023.1270296)
Supplement: Supplementary file 2 [file Table_2.pdf]

Supplementary Material

"Effectiveness of rehabilitation intervention in persons with Friedreich ataxia"

Supplementary Table 2. Comparison of the extent of improvement in adults and children.

	**Adults (N=29)**	**Children (N=13)**	***p*-value***
**SARA**	-1.12 (1.26)	-1.81 (1.33)	0.116
**FARS Total**	-3.70 (2.73)	-5.72 (5.39)	0.113
FARS LL	-0.74 (1.10)	-1.88 (1.67)	0.012
FARS UL	-1.95 (1.89)	-2.38 (2.67)	0.547
FARS US	-0.92 (1.33)	-1.33 (1.38)	0.362
**NHPT (D)**	-1.50 [-3.50, 1.30]	-4.56 [-7.80, 0.92]	0.422
**NHPT (ND)**	-1.80 [-5.77, 2.50]	-0.43 [-3.50, 2.25]	0.828
**6MWT**	28.50 [11.50, 75.00]	11.00 [0.60, 60.00]	0.430
**BBS**	3.47 (2.39)	3.56 (2.19)	0.928
**TUG**	-2.32 (4.40)	-1.56 (1.39)	0.587

Mean (SD) or Median [IQr] are shown. * P-values from: t-test (mean (SD) or Mann Whitney U test (median [IQR]). LL, Lower Limb; UL, Upper Limb; US, Upright Stability; D, dominant hand; ND, non-dominant hand
